# Versatile, Cheap, Readily Modifiable Sample Delivery Method for Analysis of Air-/Moisture-Sensitive Samples Using Atmospheric Pressure Solids Analysis Probe Mass Spectrometry

**DOI:** 10.1021/acs.analchem.2c02039

**Published:** 2022-08-05

**Authors:** Kerry A. Strong, Peter Stokes, David Parker, Amy K. Buckley, Jackie A. Mosely, Claire N. Brodie, Philip W. Dyer

**Affiliations:** † Department of Chemistry, Durham University, Durham DH1 3LE, United Kingdom

## Abstract

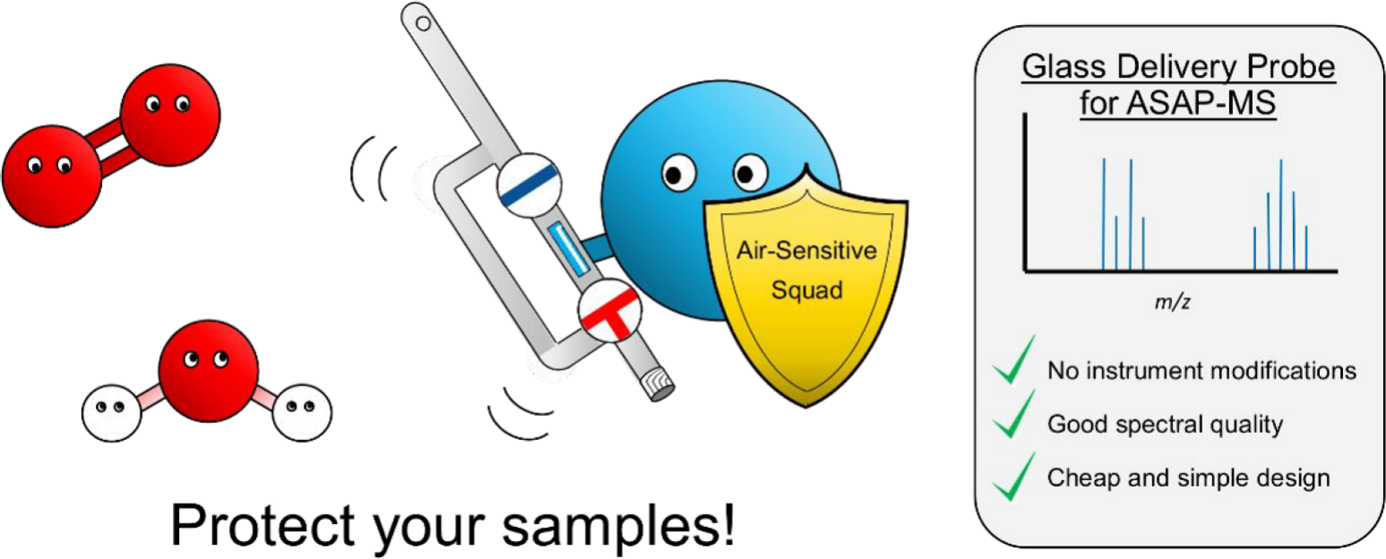

A cheap, versatile, readily modified, and reusable glass probe system enabling delivery of solid air-/moisture-sensitive samples for mass spectrometric (MS) analysis using an Atmospheric pressure Solids Analysis Probe (ASAP) is described. The simplicity of the design allows quick and easy ASAP MS analyses of sensitive solid and liquid samples without the need for any modifications to commercially available vertically loaded ASAP mass spectrometers. A comparison of ASAP mass spectra obtained for metal complexes under air and an inert atmosphere is given.

## 1 Introduction

Atmospheric pressure Solids Analysis Probe Mass Spectrometry (ASAP MS) is a direct ionization MS technique that exploits the many recent advances in ion generation at ambient pressure. This enables native sample analysis, removing the need for sample preparation through solvation or functionalization.^1^ In ASAP MS, the sample is subject to elevated temperatures in an external, heated, nitrogen-filled vaporization/ionization chamber at ambient pressure. The resulting vaporized molecules are then singly ionized (via charge transfer) before being introduced into a mass analyzer.^1^ This sequence circumvents problems often found with traditional MS techniques where ionization is achieved within the spectrometer, namely, ionization sensitivity and chemical specificity.^2,3^ Additionally, not only can ASAP MS be applied to both solid and liquid samples, it enables analysis of materials of low volatility. For example, low molecular weight oligomeric materials are increasingly being analyzed by ASAP MS in preference to other ionization techniques, such as MALDI or ESI, since using ASAP MS avoids complications that can result from the need to add a metal cation to promote ionization.^4,5^ ASAP MS has also been shown to be particularly useful in the analysis of homogeneous samples that suffer from solvent suppression (dampening of the sample signal due to interactions with the solvent) in electrospray ionization (ESI) or atmospheric pressure chemical ionization (APCI).^5,6^ Together with the features described above, the fact that the ASAP MS technique requires no prior sample preparation through dissolution in solvent makes this a particularly attractive method for the analysis of air-/moisture-/solvent-sensitive samples, with the need for rigorously dried and degassed solvents being eliminated.

While the sample analysis ionization system of a mass spectrometer is under a completely inert atmosphere (high vacuum or fed with a dry, inert gas supply), often the method by which the analyte sample is introduced necessitates brief exposure of the sample to air. This is a significant limitation for the analysis of air-/moisture-sensitive samples, and hence, a number of methods have been developed for a range of mass spectrometry (MS) ionization techniques including liquid injection field desorption ionization (LIFDI),^7,8^ electrospray ionization (ESI),^9−11^ electron impact (EI),^12,13^ and matrix-assisted laser desorption/ionization (MALDI).^14,15^ This has been extended to ASAP MS, leading to the development of so-called *i*ASAP MS, which has included the commercialization of a specialized dedicated attachment by Advion, Inc. working in collaboration with Krossing and co-workers.^16^ More recently, a related approach, “Paraffin-Inert ASAP” was been reported by Afonso, Giusti, and co-workers, which involves sealing a capillary loaded with sample with paraffin in a glovebox to protect the sample during transport to the machine.^17^ Building on our prior work around developing methods for the analysis of air-/moisture-sensitive samples via ASAP MS,^18^ we describe herein the design and use of a cheap, simple, and reusable glass probe delivery sample system whose configuration can be very easily modified to fit any vertically loading ASAP mass spectrometer providing an alternative methodology for analysis of environmentally sensitive solid and liquid materials. This approach is straightforward, readily applicable, and requires no alterations to the Waters mass spectrometry instruments used in this study and can easily be modified for use with other instruments.

## 2 Experimental Section

### Materials

[Pd(acac)_2_] (**1**) was obtained from Alfa Aesar and used without further purification. 1,2-*bis*(*di*-*tert*-butylphosphinomethyl)benzene was obtained from Lucite International and was recrystallized before use. Complexes **2**(24) and **3**(25,26) were prepared in accordance to literature procedures.

### General Considerations

Air-sensitive manipulations were carried out under an atmosphere of dry nitrogen using standard Schlenk-line and glovebox (Saffron Scientific; Innovative Technologies) techniques. Hexanes were dried using Innovative Technologies Solvent Purification System facilities. Tetrahydrofuran (THF) was dried by heating at reflux over sodium wire with a benzophenone indicator, followed by distillation. All solvents were degassed prior to use using freeze–pump–thaw techniques. ASAP (Atmospheric pressure Solids Analysis Probe) mass spectra were acquired with an LCT Premier XE mass spectrometer or Waters Xevo QToF mass spectrometer. Mass spectrometer conditions for ASAP analysis using the glass probe delivery system were optimized using a series of air-stable organometallic complexes. Details of optimization experiments can be found in the Supporting Information. Infrared spectra were recorded in the solid state using a PerkinElmer Frontier ATR-FTIR instrument.

#### Synthesis of CoBr_2_(dtbpx) (**4**)

To a solution of cobalt(II) bromide (0.27 g, 1.24 mmol) in THF (10 mL), 1,2-*bis*(di-*tert*-butylphosphinomethyl)benzene (0.50 g, 1.28 mmol) in THF (15 mL) was added dropwise. The reaction was stirred at room temperature for 16 h after which time precipitation was mediated through addition of hexanes (20 mL). The solids were collected via filtration and washed with hexanes (3 × 10 mL) before drying to yield **4** as a turquoise powder (0.69 g, 1.12 mmol, 90%) (found: C, 46.9; H, 7.15. C_24_H_44_Br_2_CoP_2_ requires: C, 47.0; H, 7.20%). MS (ASAP^+^, anaerobic) *m*/*z* 532.15 ([M-Br]^+^, 3%), 395.31 (100). IR: ν cm^–1^ 3064w (C–H), 2972–2868s (C–H), 1601w (C=C), 1472–1370s (C–H). Raman (532 nm): ν cm^–1^ 282m (Co–Br), 580s (C–C), 690m (C–P), 812m (C–C), 942m (C=C), 1027w (C–C), 1057m (C=C), 1235s (CH_2_), 1476m (CH_3_) and 1603m (C=C). Silent to ^31^P NMR spectroscopy. UV–vis (THF): λ_max_ 658, 699, and 744 nm.

### Mass Spectrometric Measurement

An open-ended glass capillary (50 mm × 1 mm) is dipped into a powdered sample to give a plug of height 1 mm and placed within the glass delivery probe (either within a glovebox or open to air depending on sample), and both PTFE taps on the glass delivery probe closed such that the sample is isolated. The probe is connected to an N_2_ gas supply set to a flow rate of 5, 10, 15, or 20 mL min^–1^ (controlled by Dywer Variable Area Flowmeter) and purged via the side arm for 20 s. The glass delivery probe is connected to an LCT Premiere XE or Waters QToF Xevo mass spectrometer through the Delrin holder to the standard ASAP adaptor and time allowed for the probe tip to reach temperature (∼1 min). Sample measurement conditions are set using MassLynx software and data collection started for a 2 or 3 min run. Here, 10–30 s into data collection, the PTFE stopcocks on the glass delivery probe are opened simultaneously to allow the capillary tube containing the sample to drop into the ionization chamber of the mass spectrometer; the sample exits the probe through the sample release hole allowing necessary ionization prior to data collection.

## 3 Results and Discussion

### 3.1 ASAP Sample Glass Delivery Probe Development

Our previous preliminary report on the analysis of air-/moisture-sensitive materials using ASAP MS demonstrated successful analysis of two highly air-sensitive materials through application of a simple inert atmosphere sample introduction method.^18^ This comprised sealing the analyte sample in a glass capillary tube inside an inert atmosphere glovebox, transporting the sealed tube to the spectrometer, and finally introducing the analyte into the spectrometer by using a rotating baffle (originally intended to divert an internal calibrant into the spectrometer) to break the sealed capillary located inside the external, heated vaporization/ionization chamber. Although simple to achieve, this approach could potentially allow fine particle debris (e.g., broken glass shards) to block the inlet or contaminate the first stages of the API interface of the mass spectrometer. This can only be limited by careful cleaning between samples, something that necessitates repeated exposure of the chamber to ambient atmosphere conditions. Additionally, this simple delivery method for the introduction of air-/moisture-sensitive samples for ASAP MS does, albeit briefly, result in the exposure of the analyte to air. For many air-/moisture-sensitive complexes, even this brief exposure is sufficient for oxidation and/or hydrolysis to occur.

The reusable sample delivery probe protocol described herein for analysis of air-/moisture-sensitive samples by ASAP MS is based upon the standard method used in vertically loaded ASAP instruments. To obtain a mass spectrum using ASAP MS for an air stable sample, a small quantity of the solid or liquid analyte is loaded onto the end of a capillary tube. This capillary is placed within a holder, which in turn is then placed within the heated vaporization/ionization chamber. This chamber is kept at slightly above atmospheric pressure with nitrogen gas and at an elevated temperature (300–650 °C). Application of a corona discharge within the chamber ionizes the nitrogen gas,^19,20^ which then transfers charge to the analyte sample, with the resulting singly charged ions then entering the sample block, extraction cone, ion optics, and finally into the TOF chamber via a sample cone (that has an applied voltage gradient).

**Figure 1:**
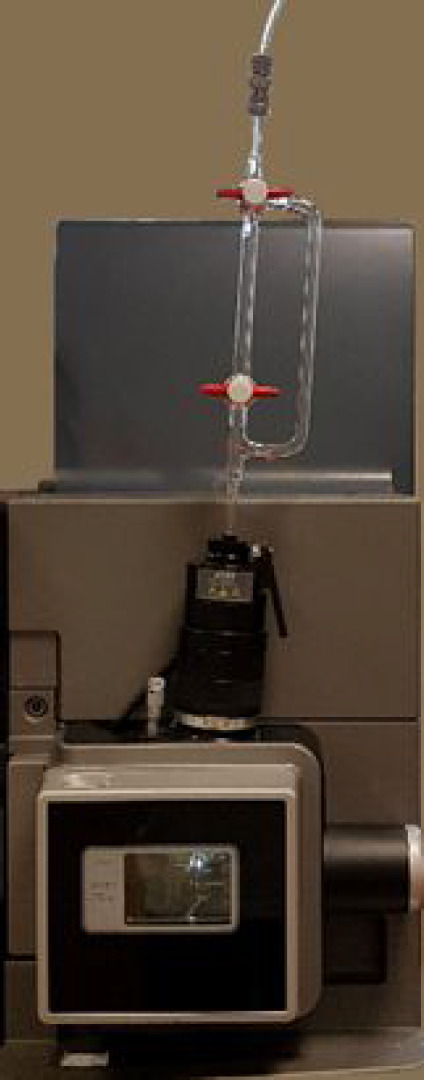
Waters Xevo QToF mass spectrometer fitted with a vertical loading ASAP inlet port with a glass sample delivery probe in position.

**Figure 2:**
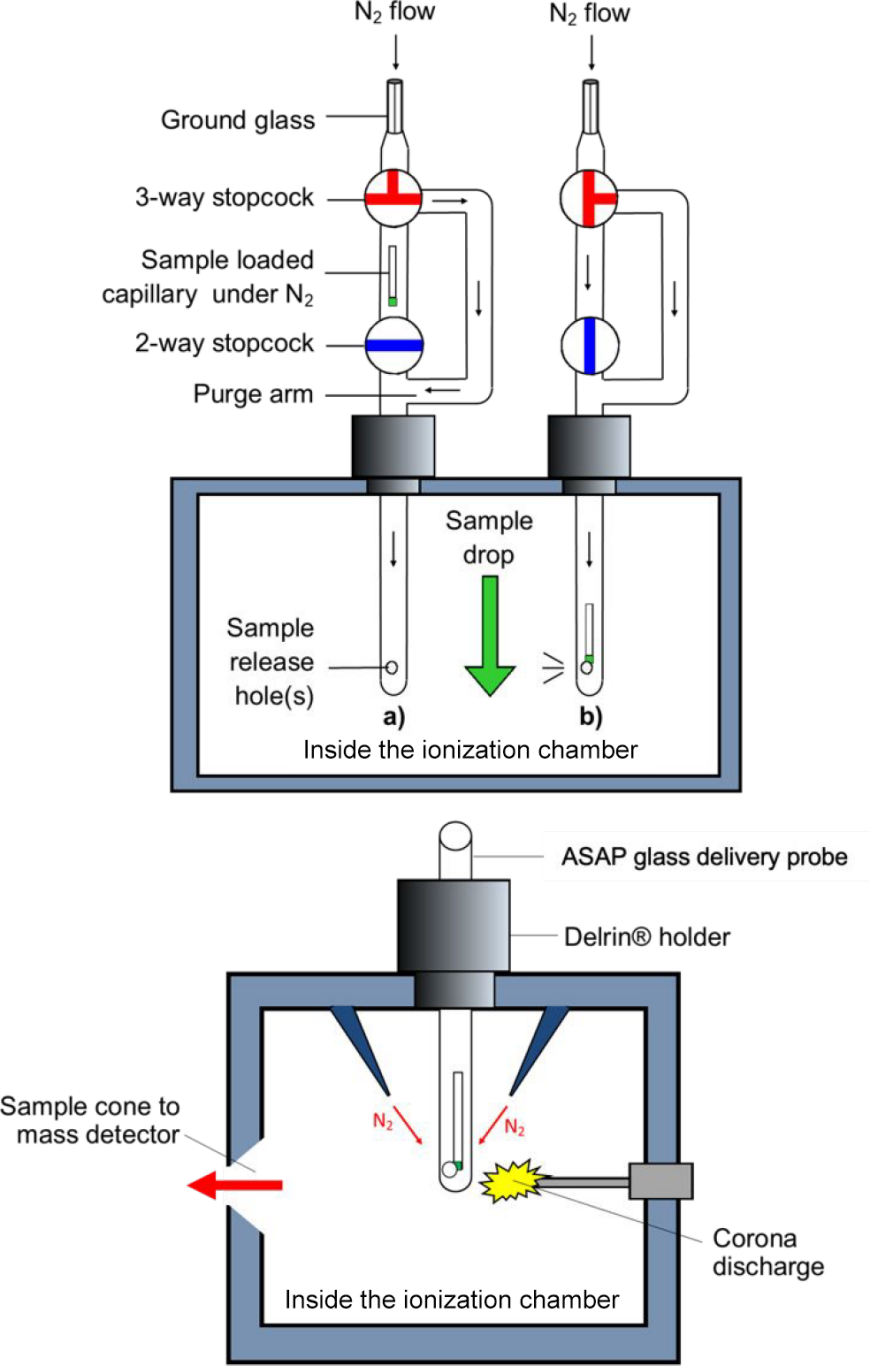
Simplified representation of ASAP glass delivery probe: (a) during purge of the probe and (b) for analysis (top). Schematic representation of the inside of the ionization chamber showing relative positions of desolvation gas (N_2_) injection system, corona discharge, and sample cone (bottom).

To circumvent sample contact with air and/or moisture, a simple low cost “glass delivery probe” was designed. This allows transportation and delivery of liquid or solid samples from a standard glovebox directly into the vaporization/ionization chamber of a standard commercial vertically loading ASAP mass spectrometer without exposure to the atmosphere (Figure 1). The necessary glass probes and Delrin probe holder were manufactured in house to specifications to fit two commercial vertically loading mass spectrometers (LCT Premiere XE ToF and Water’s Xevo QToF mass spectrometers) capable of ASAP analysis. In a glovebox, a sample-dipped glass capillary is sealed in the “glass delivery probe” under an inert atmosphere between two closed PTFE stopcocks for transportation to the mass spectrometer. The end of the “glass delivery probe” described herein is then inserted into the instrument such that the tip of the probe resides within the heated vaporization/ionization chamber. The side arm and the three-way PTFE tap arrangement allow purging of the lower end of the probe with an inert gas (Figure 2a). Subsequently, to allow sample analysis, a flow of inert gas from the top of the probe (vertical gas flow) is maintained, and both PTFE taps are opened allowing the sample-loaded capillary to drop down into the base of the probe (located within the heated zone) where the analyte is vaporized and then ionized by a corona discharge prior to entry to the TOF system as normal (Figure 2b).

The use of a reusable glass delivery probe to deliver the air-/moisture-sensitive samples into the instrument allows quick and easy ASAP mass spectrometric analysis to be performed with no alterations to existing mass spectrometers. Using this methodology sample turnaround is only limited by cleaning/drying of the probe(s) between each use.

### 3.2 Sample Introduction: Comparison of Capillary vs Glass Delivery Probe ASAP Methods

**Figure 3:**
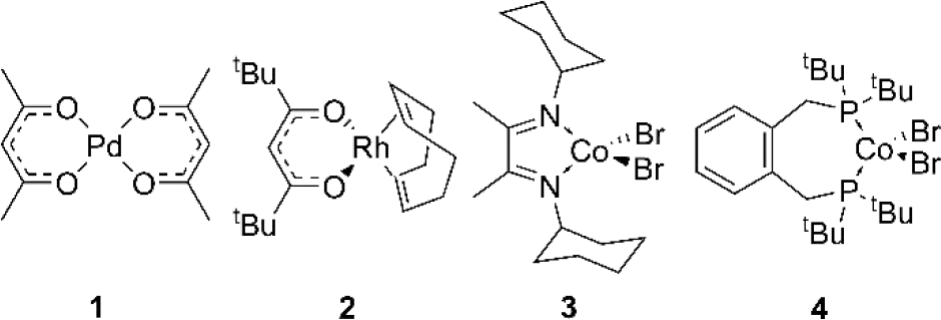
Representative coordination and organometallic complexes (**1**–**4**) tested for analysis by ASAP MS using capillary or glass probe delivery methods.

In the first instance, the air-stable coordination and organometallic complexes [Pd(acac)_2_] (**1**) and [Rh(COD)(tmh)] (COD = 1,5-cyclooctadiene, tmh = 2,2,6,6-tetramethyl-3,5-heptanedionato) (**2**), as well as moisture-sensitive [CoBr_2_(ADI^Cy^)] (**3**) (ADI^Cy^ = *N,N*-bis(cyclohexyl)-1,2-diimino-1,2-dimethylethane) and air- and moisture-sensitive [CoBr_2_(dtpbx) (**4**) (dtbpx = 1,2-bis(di-*tert*-butylphosphinomethyl)benzene) (Figure 3) were selected to allow direct comparison between traditional (where the sample is exposed to the atmosphere), capillary-loaded, and “glass delivery probe”-loaded ASAP MS spectra. These samples were also employed in mass spectrometer optimization experiments. In all the following ASAP MS experiments where the glass delivery probe is applied, the probe was allowed to “warm up” for 30 s after introduction to the ionization chamber before the sample is “dropped”.

**Figure 4:**
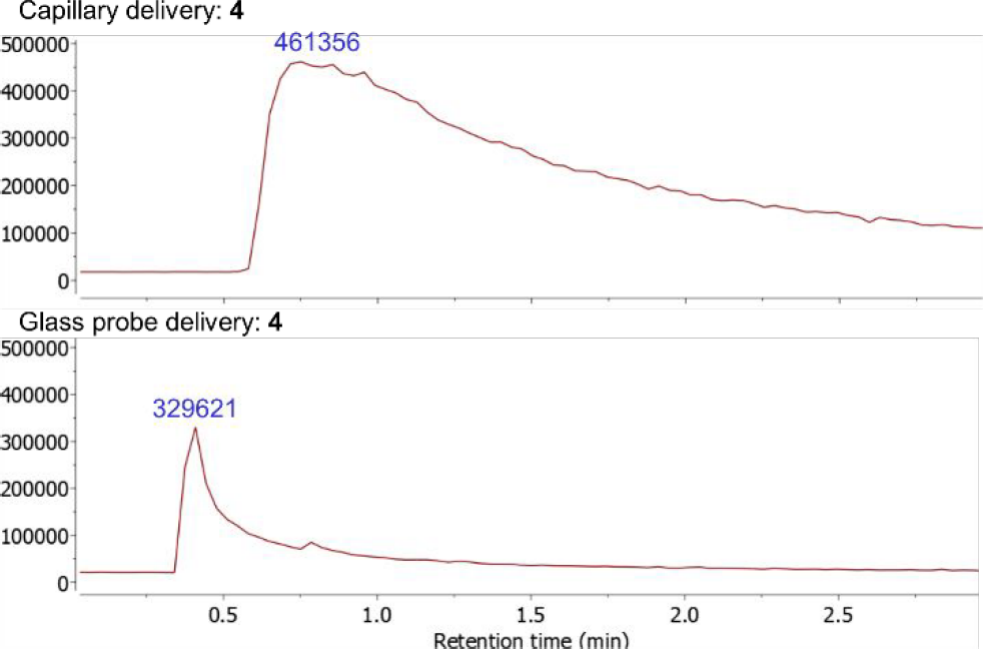
Comparison of total ion count (TIC) signals obtained using ASAP (top) and glass delivery probe ASAP (bottom) MS techniques with a sample of [CoBr_2_(dtbpx)] (**4**).

The original design of the “glass delivery probe” included a single sample release hole positioned so as to face the sample cone (i.e., ToF system inlet) to maximize the amount of sample being introduced into the ToF analyzer (Figure 2a, b; top). However, this arrangement results in the corona discharge pin facing glass, which is found to limit the intensity of the resulting signal. We hypothesized that introduction of a second sample release hole in the probe tip would provide a greater means of escape for the vaporized sample as a result of direct impingement of the corona discharge upon the sample within the probe and hence greater MS signal intensity. However, no significant increase to the signal intensity in the total ion count (TIC) measured for a sample of **3** ([M–CoBr_2_]^+^ signal) was observed moving from one to two sample release holes (cf. TIC intensity ∼20,000 and ∼12,000 for one and two sample release holes, respectively). It is important to note that in all the ASAP MS experiments undertaken using the glass delivery probe method, the TIC signal intensity obtained was significantly lower than that obtained when using the traditional dipped capillary-loaded ASAP MS (Figure 4), but the TIC signal intensity remained at a level that did not impact on analysis performance. Indeed, this reduction to the intensity in the signal observed may be an advantage due to reducing the likelihood of overloading the MS ion detector.^21^

**Figure 5:**
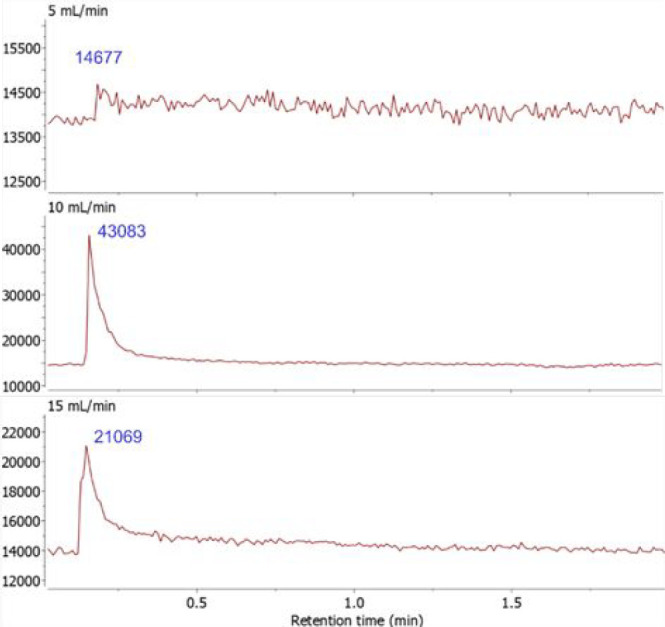
Comparison of total ion count (TIC) signals using the glass delivery probe ASAP MS technique with various vertical N_2_ gas flow rates at 450 °C from a sample of [CoBr_2_(ADI^Cy^)] (**3**): top, 5 mL/min; middle, 10 mL/min; bottom, 15 mL/min vaporization/ionization chamber.

The signal attenuation found when using the glass delivery probe ASAP MS arrangement has been attributed to a cooling effect caused by the “sweep”/purge gas flow applied to the probe, which results in less efficient volatilization of the solid sample in the ASAP chamber. For example, at a high (15 mL/min) purge gas flow rate, a significantly reduced TIC signal intensity is measured compared to that observed with a gas purge flow rate of 10 mL/min due to this cooling effect (Figure 5). However, further reduction to the purge gas flow rate to 5 mL/min shows an attenuation in TIC signal intensity, suggesting that the purge gas flow also aids release of the sample to the ionization chamber where it can then be vaporized and analyzed (characterized by a sharp TIC signal with high intensity immediately upon sample introduction).

Physical sample ejection from the probe/capillary is further inferred from the observation of sprayed/deposited samples upon the walls, door, and sample cone of the vaporization/ionization chamber post analysis when very high (>15 mL/min) purge gas flow rates are used, which resulted in the bulk of the sample being outside of the main heated zone and thus not undergoing efficient vaporization. Thus, a moderate purge gas flow rate is therefore optimal, such that cooling effects are minimized, and gas-entrained physical sample ejection from the probe is enhanced. Notably, however, the signal intensity of MS TIC obtained when using the glass probe delivery system can be increased to an intensity comparable to that of a sample run under standard ASAP conditions (loaded capillary) simply by increasing the temperature of the vaporization gas within the purge gas flow. This temperature increase combats the cooling effects of the purge gas flow. Varying the sample cone voltage used for both traditional capillary-loaded and “glass delivery probe” ASAP MS experiments was found to have an identical and significant impact upon the fragmentation observed within the resulting mass spectra obtained as reported previously.^22^ Thus, standard cone voltages (e.g., 30 V) were used for all experiments.

**Figure 6:**
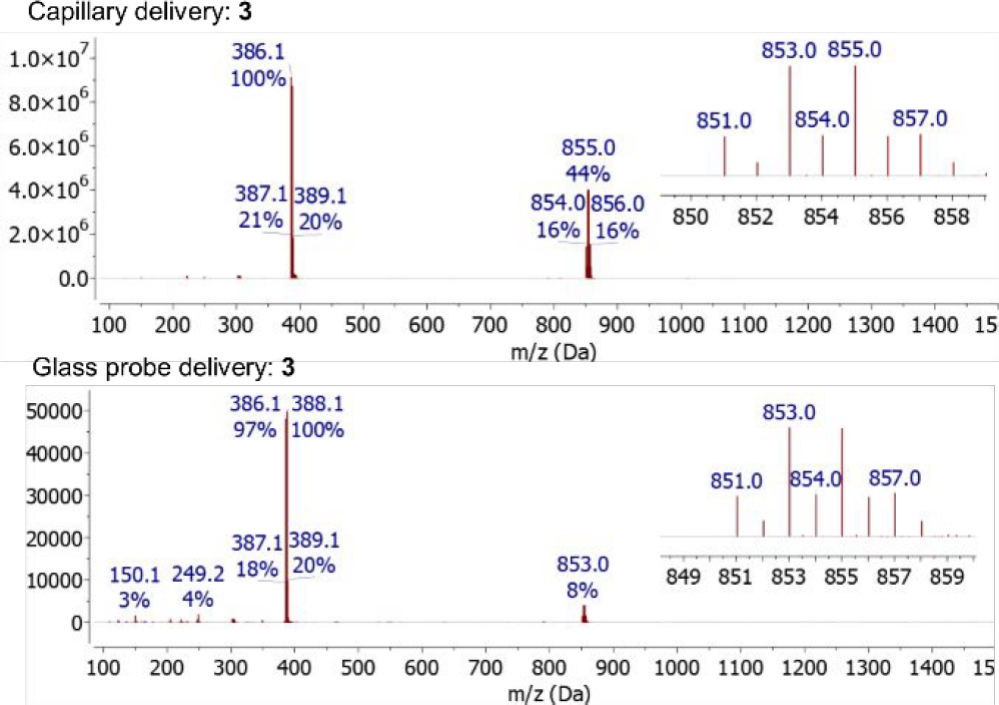
Comparison of the ASAP mass spectra obtained for a sample of [CoBr_2_(ADI^Cy^)] (**3**) using a traditional dipped capillary (450 °C; top) and glass sample delivery probe (500 °C; N_2_ flow rate = 10 mL/min; bottom) sample introduction methods. For both ionization methods, *m*/*z* 851.0 corresponds to [2M–Br]^+^. Insets show experimental isotope patterns.

The ASAP mass spectra obtained following introduction of the sample into the spectrometer using the glass delivery probe under anaerobic conditions are comparable to those obtained with standard dipped capillary loading. For example, Figure 6 compares the spectra obtained from complex **3** with dipped capillary and glass delivery probe sample loading methods. Of note, both techniques show the [M–Br]^+^ ion as the highest intensity (100%) ion present. In addition, the spectra resulting from both loading techniques show some recombination of ions within the mass spectrometer generating the ion corresponding to [2M–Br]^+^ at *m*/*z* 851.0 Da (Figure 6), as a result of a high concentration of sample. When the glass delivery probe sample introduction method is used to load the sample, the relative intensity of this dimer (recombination) product (compared to the [M–Br]^+^ ion) produced within the mass spectrometer is reduced relative to when the sample is introduced using the dipped capillary approach (cf. 16% with dipped capillary and 3% with glass delivery probe). It is proposed that this difference is the result of a smaller amount of sample being released into the ion source region (as indicated by a reduced TIC) when the glass probe delivery method is used, vide supra. It is likely that lower sample concentration will result in a corresponding reduction in the production of in-source recombination products such as [2M–Br]^+^ observed for complex **3**, an effect that has been observed upon reducing sample concentration during ESI-MS analysis.^23^

### 3.3 ASAP MS of an Air-Sensitive Sample

**Figure 7:**
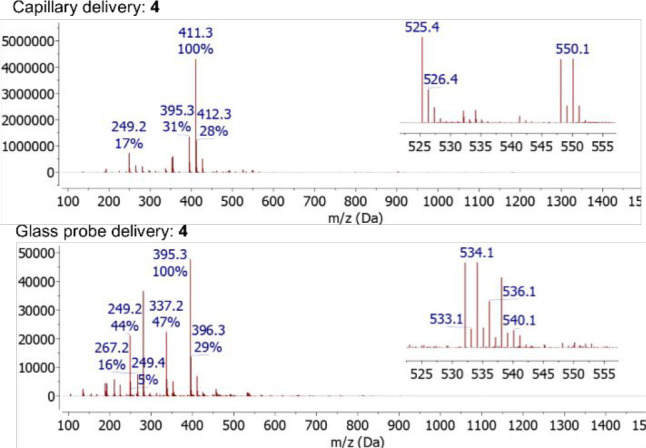
Comparison of the ASAP mass spectra obtained from a sample of an as-obtained reaction mixture (solid state) containing [CoBr_2_(dtbpx)] (**4**) introduced via a standard dipped capillary (top), *m*/*z* 411.3 Da corresponds to [M–CoBr_2_+OH]^+^, and using a glass sample delivery probe (bottom), *m*/*z* 395.3 Da corresponds to [M–CoBr_2_+H]^+^. Insets show experimental isotope patterns.

Having demonstrated that mass spectra of comparable quality could be obtained using both standard dipped capillary and glass delivery probe loading during ASAP MS experiments, the glass delivery probe was tested for the ASAP MS analysis of air-sensitive samples. As an example of the benefits of the glass probe-loaded sample over traditional capillary loading, the mass spectra obtained of an air-sensitive, reaction product mixture (solid state) in which the predominant species is [CoBr_2_(dtbpx)] (**4**) (as determined by Raman, UV–vis, and NMR spectroscopies) using both sample introduction methods are shown in Figure 7.

From the ASAP MS spectrum obtained using the glass sample delivery probe, there is a significantly lower degree of oxidation of the diphosphine (dtbpx) (cf. 15% vs 70% oxidation of [M-CoBr_2_]^+^) observed as shown by the highest intensity ion at *m*/*z* 395.3 Da, which corresponds to [M–CoBr_2_+H]^+^. Here, the strong signal associated with this ligand results from the ionization process, as no free phosphine is observed in the corresponding ^31^P NMR spectrum (THF) obtained of this crude mixture. In contrast, the ASAP MS spectrum obtained from the reaction sample introduced using a dipped capillary shows the highest intensity ion as being the partial oxidation product [M–CoBr_2_+OH]^+^ at *m*/*z* 411.3 Da. Further, use of the glass delivery probe allows observation of the [M–Br]^+^ ion of [CoBr_2_(dtbpx)] (**4**) at *m*/*z* 532.1 Da within the resulting ASAP-MS spectrum, whereas this ion signal is very weak when the same sample is analyzed using the dipped capillary technique (Figure 7). It is also noted that use of the dipped capillary gives rise to an additional, but weak, signal corresponding to [M–Br+O]^+^ at *m*/*z* 548.1 Da (calc. *m*/*z* 548.1 Da). Irrespective of the sample introduction method, the expected molecular ion (*m*/*z* 611.1 Da) arising from ionization of **4** is not observed.

## 4 Conclusions

This work describes a very cheap, straightforward, easily manufactured and modified, reusable delivery system to enable the analysis of air-/moisture-sensitive samples by ASAP MS. This method can be viewed as an alternative to *i*ASAP methods reported previously.^16−18^ The use of a simple “glass probe” to transport and deliver samples from a glovebox to an ASAP-capable mass spectrometer avoids the need to either house an MS instrument or MS sample introduction inlet in a glovebox while maintaining inert conditions and is beneficial in terms of cost (cf. glovebox-coupled MS^9,10^), does not require deconvolution of the resulting spectra (cf. paraffin inert ASAP–piASAP^17^), and removes the risks associated with broken glass fragments collecting in the mass spectrometer source (cf. our previous preliminary report on air-sensitive ASAP MS^18^).

It was found that a higher temperature is required to achieve satisfactory vaporization of the coordination and organometallic complexes tested for ASAP MS analysis when using the glass probe delivery system (450–550 °C) compared to traditional dipped capillary ASAP MS analysis (350–450 °C). This is necessitated by the need to compensate for the cooling effect of the flow of nitrogen purge gas through the glass probe into the ionization chamber. The TIC in mass spectra generated while using the glass delivery probe is lower than that obtained under standard ASAP conditions. However, the signal obtained when the glass delivery probe is employed remains sufficient for confident mass spectral assignments to be made. The ASAP mass spectra obtained when using the glass delivery probe are comparable to those obtained using a capillary-loaded ASAP MS technique.

Further studies are underway and are investigating preheating of the inert gas used within the “sweep”/purge gas flow to reduce the observed cooling effect and the subsequent decrease in TIC. In addition, the anaerobic sample delivery technique described herein does not currently offer the option of high-resolution mass determination (Da, 4 d.p.) due to the aqueous-based lock-mass solutions applied within Waters mass spectrometers. Work is currently ongoing to investigate suitable compounds for use in anaerobic MS to allow high resolution mass determination of air-/moisture-sensitive samples.

## Supporting Information Available

Details of the glass sample delivery probe specifications and design including technical drawing and mass spectrometric data and optimization (PDF)
