# Supplementary material for: Versatile, Cheap, Readily Modifiable Sample Delivery Method for Analysis of Air-/Moisture-Sensitive Samples Using Atmospheric Pressure Solids Analysis Probe Mass Spectrometry
Source: Anal Chem. 2022 Aug 5;94(32):11315–20. doi: 10.1021/acs.analchem.2c02039 (PMC9386681; doi:10.1021/acs.analchem.2c02039)
Supplement: Supplementary file 1 — ac2c02039_si_002.pdf [file ac2c02039_si_002.pdf]

Supporting Information

Versatile, Cheap, Readily Modifiable Sample Delivery Method for Analysis of Air-/Moisture-Sensitive Samples Using Mass Spectrometry

Kerry A. Strong,[a] Peter Stokes,[a] David Parker,[a] Amy K. Buckley,[a] Jackie A. Mosely,[a][b] Claire N. Brodie*[a][c] and Philip W. Dyer*[a]

[a] Department of Chemistry, Durham University, South Road, Durham, DH1 3LE, UK.

[b] Current Address: School of Health and Life Sciences, Teesside University, Middlesbrough, UK.

[c] Current Address: Department of Chemistry, University of York, YO10 5DG, UK.

*Email: claire.brodie@york.ac.uk; +44 (0)1904 322536, p.w.dyer@durham.ac.uk; +44 (0) 191 33 42150

Table of Contents

1.1 Inert atmosphere ASAP glass sample delivery probe design: S2

1.2 Mass Spectra of Complexes **1** and **2**.. S3

1.3 Summary of key mass spectrometric data S6

1.1 Inert atmosphere ASAP glass sample delivery probe design:

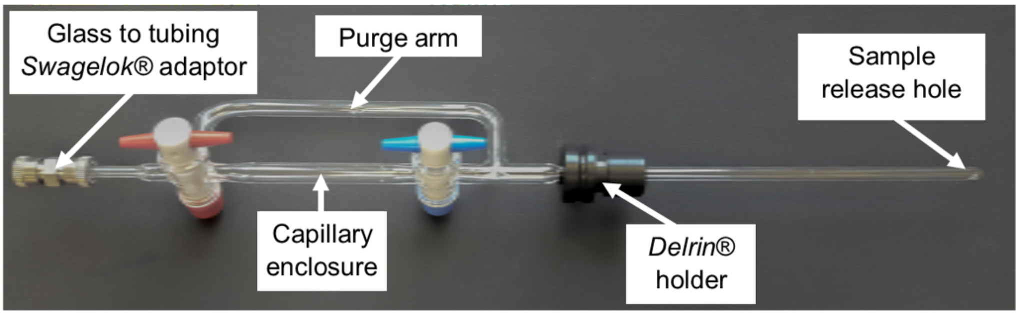

Figure S1 Glass sample delivery probe design for ASAP Mass Spectrometric experiments performed under an inert atmosphere fitted with *Delrin*® holder and glass to tubing Swagelok® adapter.

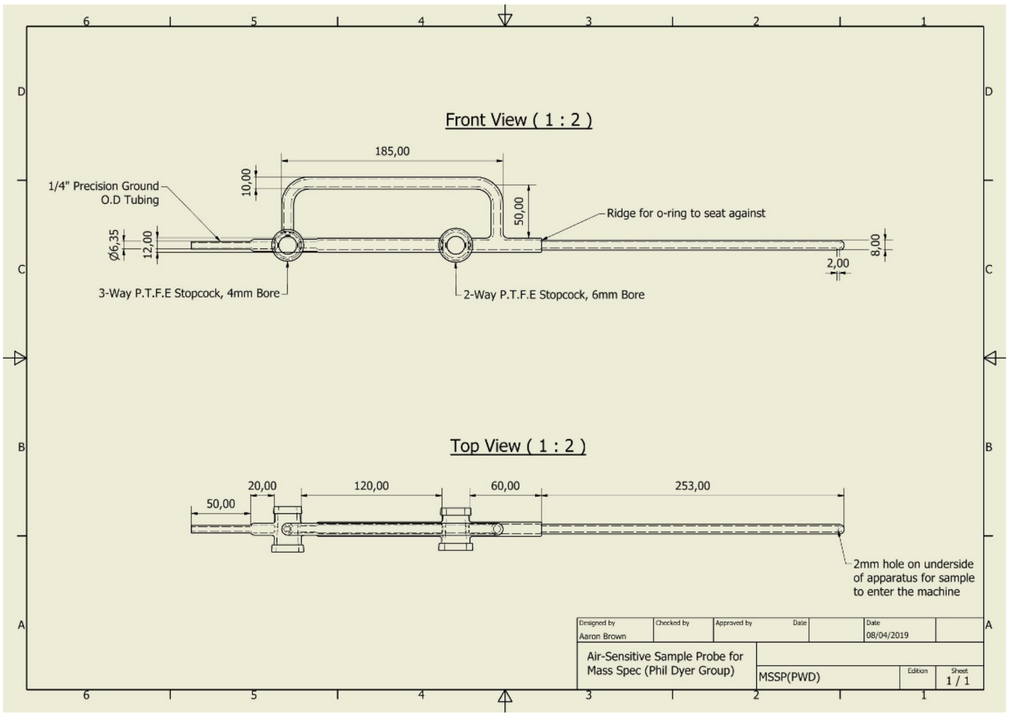

Figure S2 Technical rendering of glass probe design for glass sample delivery probe assembly for ASAP Mass Spectrometric experiments performed under an inert atmosphere. Dimensions shown are appropriate for an *LCT Premiere XE Mass Spectrometer* fitted with vertical ASAP adapter.

1.2 Mass Spectra of Complexes 1 and 2

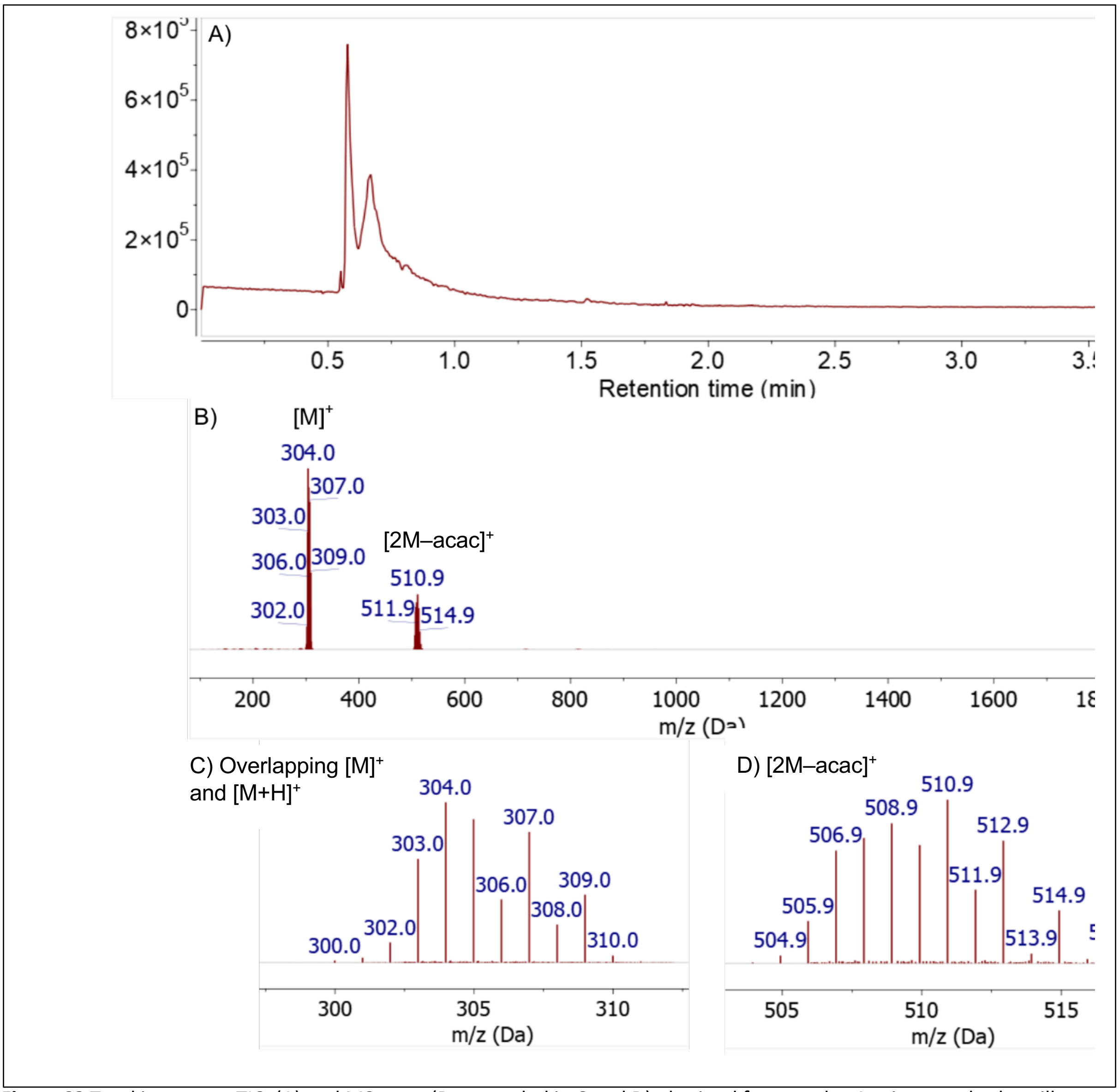

Figure S3 Total ion count, TIC, (A) and MS trace (B; expanded in C and D) obtained for complex **1** using standard capillary to introduce sample (see Table S1, Entry 2 for details). Note, from the relative intensities of the peaks C) corresponds to an overlapping of the isotope patterns for the $[M]^+$ and $[M+H]^+$ ions (see calculated isotope patterns in **Figure S4**).

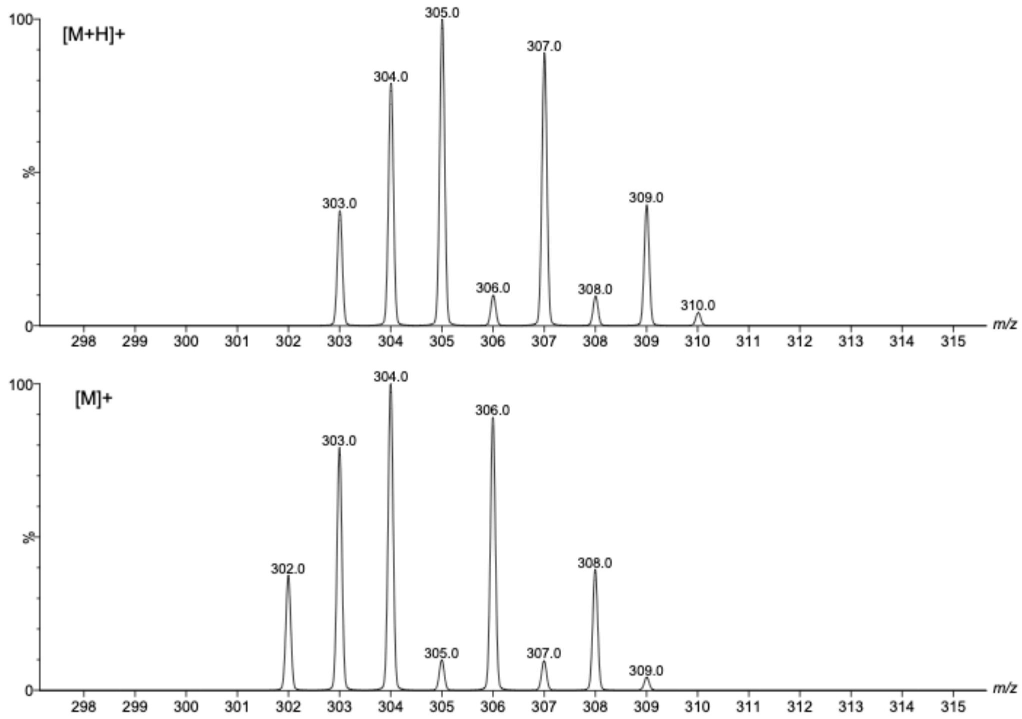

Figure S4 Calculated isotope patterns for the $[M+H]^+$ and $[M]^+$ ions of complex **3**.

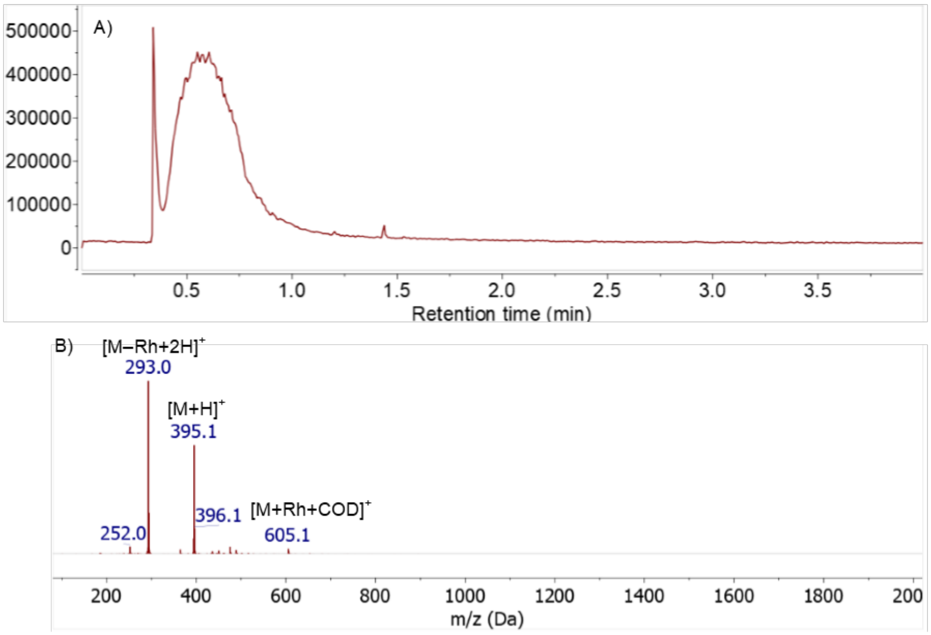

Figure S5 Total ion count, TIC, (A) and MS trace (B) obtained for complex **2** using glass sample delivery probe to introduce sample (see table S1, Entry 5 for details).

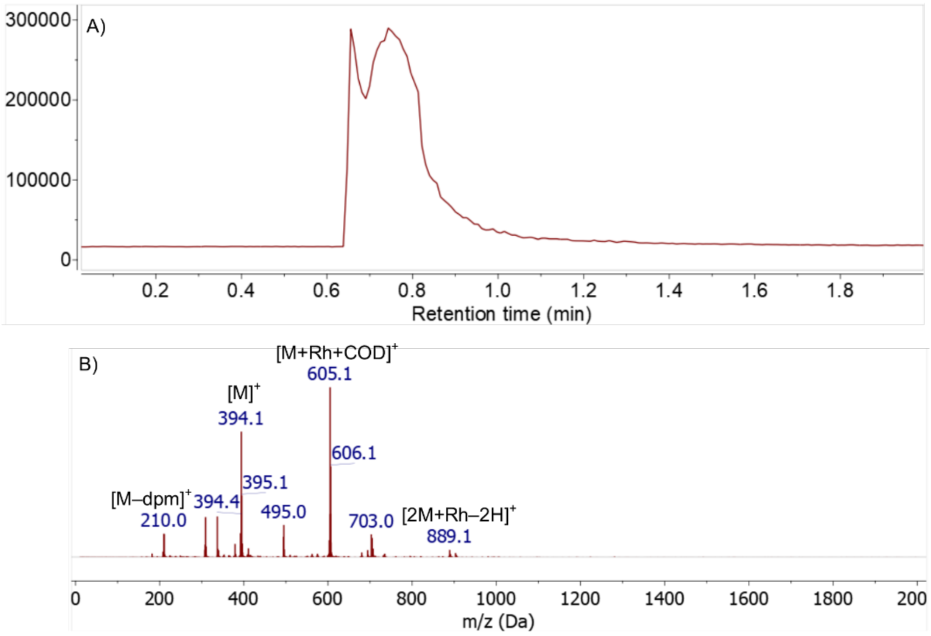

Figure S6 Total ion count, TIC, (A) and MS trace (B) obtained for complex **2** using standard capillary to introduce sample (see table S1, Entry 9 for details).

1.3 Summary of key mass spectrometric data

Table S1 Summary of the ASAP optimisation experiments with observed mass fragments and intensities shown. Where DS = desolvation gas temperature, cV = cone voltage, VF = vertical flow rate, SB = Source block temperature, PD = probe design (1 or 2 denotes number of sample holes and cc and oc refer to the use of an 'open-ended' (oc) or 'closed' (cc) capillary within the probe).

Entry	Sample*	Instrument	DS /°C	cV /V	VF /mL min^{-1}	SB /°C	PD	**Fragments observed:** %observed (mass /Da)					
								**M/M+H**	**Pd_2L_3**	**Pd_2L_4**	**Pd_2L_6**		
1	**1**	*LCT* ToF	300	30	N/a	120	N/a	100 (304.7)	91 (510.5)	12 (609.4)	12 (814.2)		
2			350					100 (304.0)	14 (510.9)				
3			400					100 (305.0)	4 (510.9)				
								**M+H**	**M+Rh+COD**	**dpm+2H**	**M−Rh+2H**		
4	**2**	*LCT* ToF	300	30	n/a	120	1 - cc	100 (395.1)	50 (605.1)		7 (293.1)		
5			350					75 (395.1)	3 (605.1)	7 (185.2)	100 (293.0)		
6			400					68 (395.1)	100 (605.1)				
								**M/ M+H**	**M+Rh+COD**	**M−dpm**			
7	**2**	*Xevo* QToF	350	15	N/a	120	N/a	84 (394.1)	100 (605.1)				
8				20				88 (394.1)	100 (605.1)				
9				30				56 (394.1) /76 (395.1)	100 (605.1)	29 (211.0)			
10			450	20				96 (394.1)	100 (605.1)	2 (211.0)			
								**M**	**Pd_2L_3**	**PdL**	**PdL+O**	**Pd_2L_2−H**	**Pd_2L_2+O**
11	**1**	*Xevo* QToF	350	15	N/a	120	N/a	97 (304.0)	100 (511.0)	28 (204.9)	4 (221.0)	4 (408.9)	6 (424.9)
12				20				59 (304.0)	100 (510.9)	40 (204.9)	10 (221.0)	8 (408.9)	15 (424.9)
13				20		150		67 (304.0)	100 (510.9)	40 (204.9)	8 (221.0)	9 (408.9)	16 (424.9)
14			450	20		120		16 (304.0)	100 (510.9)	8 (204.9)	2 (221.0)	11 (408.9)	18 (424.9)
15				30				34 (304.0)	100 (510.9)	68 (204.9)	19 (221.0)	47 (408.9)	60. (424.9)
16				40				Almost every O/C fragment possible					
								**M−Br (386.1)**	**$Co_2L_2Br_3$ (851.1)**				
17	**3**	*Xevo* QToF	450	30	5	120	1 - oc	97 (386.0)	5 (851.0)				
18					10			100 (386.0)	21 (851.0)				
19			350		5								
20			400					100 (386.0)					
21					10			100 (386.0)	8 (851.0)				
22					15			96 (386.0)	3 (851.0)				
23			450		10		1 - cc						

*Samples used: [Pd(acac)$_2$] (**1**), [(COD)Rh(tmh)] (**2**) and [$CoBr_2$(ADI^{Cy})] (**3**); tmh = 2,2,6,6-tetramethyl-3,5-heptanedionato; ADI^{Cy} = *N,N*-bis(cyclohexyl)-1,2-diimino-1,2-dimethylethane.

Table S1: continued.

Entry	Sample*	Instrument	DS /°C	cV /V	VF /mL min^{-1}	SB /°C	PD	**Fragments observed:** %observed (mass /Da)			
								**dba+H**			
24	**5**[a]	*LCT* ToF	400	30	N/a	120	N/a	100 (235.1)			
25			450	30				100 (235.1)			
26				40				100 (235.1)			
27				50				100 (235.0)			
28				60				100 (235.1)			
								**M–Br (386.1)**	**$Co_2L_2Br_3$ (851.1)**	**M (465.0)**	**M–H (464.0)**
29	**3**	*Xevo* QToF	450	30	N/A	140	N/A	100 (386.1)	16 (851.031)		
30					10		2 - oc	100 (386.1)			
31								100 (386.1)			
32					15			100 (386.1)			
33			500		10			100 (386.1)	3 (851.0)		
34			550					100 (386.1)	3 (851.0)		1 (464. 0)
35			600					100 (386.1)	22 (851.0)		
36			450		5			100 (386.1)	1 (851.0)		
37					0			100 (386.1)	3 (851.0)		
38			550		10		2 - oc	100 (386.1)			
39			450		0			100 (386.1)	3 (851.0)		
40					5			100 (386.1)			
41			500					100 (386.1)	3 (851.0)		
42			550					100 (386.1)	1 (851.0)		
43			600					100 (386.1)			
44			450		0		2 - oc	100 (386.1)	1 (851.0)		
45			500					100 (386.1)	3 (851.0)		
46			600		10			100 (386.1)	4 (851.0)	1 (462.0)	1 (464.0)
46-LM							N/A	100 (386.1)	26 (851.0)		

*Samples used: $[Pd(acac)_2]$ (**1**), [(COD)Rh(tmh)] (**2**) and $[CoBr_2(ADI^{Cy})]$ (**3**); and $[Pd_2(dba)_3]\cdot CHCl_3$ (**5**); tmh = 2,2,6,6-tetramethyl-3,5-heptanedionato; ADI^{Cy} = *N,N*-bis(cyclohexyl)-1,2-diimino-1,2-dimethylethane. [a]not included in main discussion due to poor volatility for ionisation.
